# RETRACTION: KIF4A Regulates the Progression of Pancreatic Ductal Adenocarcinoma through Proliferation and Invasion

**DOI:** 10.1155/bmri/9759616

**Published:** 2025-04-29

**Authors:** BioMed Research International

**RETRACTION**: J. Chen, C-C. Zhao, F-R. Chen, G-W. Feng, F. Luo, and T. Jiang. “KIF4A Regulates the Progression of Pancreatic Ductal Adenocarcinoma through Proliferation and Invasion,” *BioMed Research International* no. 2021 (2021): 8249293–12. https://doi.org/10.1155/2021/8249293.

The above article, published online on 11 November 2021 in Wiley Online Library (wileyonlinelibrary.com), has been retracted by agreement between the Section Editor of the journal Gerald Brandacher; and John Wiley & Sons Ltd.

The retraction has been agreed following an investigation of the concerns raised by *Hoya camphorifolia* on PubPeer [1], which identified multiple instances of inappropriately overlapping figures.

Specifically:

-Figure 4a: The two images of the BxPC-3 cell cultures (right) are identical to the 2 images of KTC-1 cultures shown in Figure 3a of [2] after a horizontal flip.

-Figure 6a: The first and third tumors in the top row appear identical to the first and fourth tumors in the bottom row in Figure 5a of [3].

As a result of the investigation, the data and conclusions of this article are considered unreliable.

Tao Jiang and Jing Chen disagree with the retraction. All other authors have been informed of this decision but did not provide a response.
